# Research landscape of precision medicine in cardiology

**DOI:** 10.6026/973206300211886

**Published:** 2025-07-31

**Authors:** Shrinidhi Sivasubramaniam, Keerthika Muniasamy, Elakkiya L., Sanjit Pradeep, Rohan D., Yogesh S., Sree Nithish Kanna Baskaran, Kaarthik Akash Srikanth

**Affiliations:** 1 Institute of Internal Medicine, Madras Medical College, Chennai, India; 2 Department of Surgery, Madras Medical College, Chennai, India; 3 Department of Internal Medicine, Sri Ramachandra Institute of Higher Education and Research, Chennai, India; 4 Institute of Internal Medicine, Madras Medical College and RGGGH, Chennai and MHPE SCHOLAR, Sri Balaji Vidyapeeth, Pondicherry, India; 5 Duty Medical Officer, ICU, SRM Global Hospitals, Kattankulathur, Tamil Nadu, India; 6 Department of Internal Medicine, Thanjavur Medical College, Thanjavur, India

**Keywords:** Precision cardiology, genomic medicine, pharmacogenomics, biomarkers, machine learning, personalized therapy, cardiovascular phenotyping

## Abstract

Precision medicine in cardiology integrates genomic, proteomic, and phenotypic data to tailor prevention, diagnosis, and treatment strategies for individual patients. Recent advances have identified genetic variants associated with arrhythmias, cardiomyopathies, and coronary artery disease, enabling risk stratification and targeted therapies. High-throughput "omics" technologies and machine-learning algorithms have facilitated the discovery of novel biomarkers and have refined phenotypic subgroups within heterogeneous cardiac disorders. Pharmacogenomic profiling has demonstrated potential to optimize drug selection and dosing, reducing adverse events and improving therapeutic efficacy. Despite these breakthroughs, challenges such as data integration, clinical implementation barriers, and ethical considerations remain. Ongoing efforts in large-scale consortia, real-world data registries, and adaptive clinical trial designs are expanding the evidence base. This review synthesizes the current research landscape, highlights emerging technologies, and discusses future directions for personalized cardiovascular care.

## Background:

Cardiovascular diseases remain the leading cause of morbidity and mortality worldwide, accounting for nearly 18 million deaths annually and imposing substantial economic and social burdens on healthcare systems. Traditional management paradigms predicated on population-based risk factors such as hypertension, hyperlipidemia, and diabetes have yielded significant gains in prevention and treatment, yet inter individual variability in disease presentation and therapeutic response persists [1]. For example, only subsets of patients with similar clinical profiles derive maximal benefit from standard heart failure regimens or antiplatelet therapy, while others experience suboptimal outcomes or adverse drug reactions. Precision medicine seeks to transcend this one-size-fits-all" model by leveraging patient-specific molecular and phenotypic data to inform risk stratification, diagnostic accuracy, and therapeutic choice. Technological advances over the past decade most notably next-generation sequencing, high-throughput proteomics, and single-cell transcriptomics have enabled comprehensive characterization of genetic variants, protein expression patterns, and cellular phenotypes that drive cardiovascular pathology [2]. These insights have identified pathogenic mutations in genes such as LMNA and MYH7 underlying familial cardiomyopathies, elucidated polygenic risk scores correlating with atherosclerotic burden, and uncovered novel biomarkers (*e.g.*, microRNAs, circulating endothelial cells) predictive of myocardial injury and remodeling. Complementing molecular profiling advanced computational tools including machine-learning algorithms and network-based analyses integrate multi-modal datasets (genomic, proteomic, imaging and electronic health records) to delineate discrete patient subgroups and predict individual trajectories [3]. Such approaches have refined phenotypic classifications of heart failure beyond left ventricular ejection fraction, unmasking biologically distinct endotypes with divergent prognoses and treatment sensitivities. Moreover, pharmacogenomic insights exemplified by CYP2C19 genotyping to guide clopidogrel and SLCO1B1 testing to mitigate statin-induced myopathy have demonstrated the capacity to reduce adverse events and optimize drug efficacy in routine practice [4]. Despite these advances, significant barriers hinder the translation of precision cardiology into widespread clinical adoption. Challenges include the integration and standardization of heterogeneous data sources, the high costs and logistical complexities of multi-omics assays, ethical and regulatory considerations surrounding genetic information, and persistent disparities in access to genomic testing [5]. In response, large international consortia and real-world registries have been established to harmonize data collection, validate computational models across diverse populations, and evaluate cost-effectiveness. This narrative review will map the current research landscape of precision medicine in cardiology highlighting key discoveries, technological enablers, and critical implementation challenges to inform future directions in personalized cardiovascular care.

## Literature search strategy:

A comprehensive literature search was undertaken to identify key advances in precision cardiology from January 2010 through June 2025 by querying PubMed/MEDLINE, Embase, and Scopus using both controlled vocabulary (*e.g.*, MeSH terms such as "precision medicine" and "personalized cardiology") and free text keywords (including "genomics," "proteomics," "pharmacogenomics," "polygenic risk score," "machine learning," and "multi omics") [6]. After removing duplicates, titles and abstracts were screened to exclude non English articles, conference abstracts without full data, case reports, and studies lacking primary translational or clinical outcomes. Full texts of the remaining articles were reviewed for original research and comprehensive reviews that detailed molecular profiling in cardiac diseases, development or validation of computational risk models, clinical implementation of pharmacogenomic strategies, and identification of novel biomarkers or imaging phenotypes linked to personalized interventions [7]. Data were extracted on study design, patient cohorts, omics platforms or analytical methods, principal findings regarding genetic variants or molecular signatures, clinical endpoints such as event rates and drug response, and reported barriers to implementation in practice. Reference lists of included papers were hand searched to capture additional seminal works. Although this narrative review does not follow formal systematic review protocols, the transparent, reproducible search and selection process provides a representative overview of the evolving research landscape in precision medicine for cardiology [8].

## Overview of mechanisms and modalities:

Precision cardiology uses a range of molecular and computational techniques to address the heterogeneity of cardiovascular disease and tailor interventions to individual patients. Central to this approach are high throughput genomic assays, such as whole exome and whole genome sequencing, which reveal both common and rare variants that influence susceptibility to cardiomyopathies, arrhythmias and atherosclerotic disorders [9]. These genetic data can be combined into polygenic risk scores that the effects of many loci to stratify individuals by their risk of myocardial infarction or heart failure. In addition to DNA analysis, transcriptomic and proteomic profiling of myocardial tissue, blood cells and circulating plasma proteins has identified dysregulated pathways in inflammation, extracellular matrix remodeling and metabolism that define distinct disease subtypes and predict adverse outcomes [10]. Advanced imaging modalities, such as strain based echocardiography, cardiac magnetic resonance tissue characterization and positron emission tomography, provide detailed measures of function, structure and metabolism *in vivo*. When these imaging biomarkers are combined with molecular data, they improve patient subgroup definitions and help predict response to therapies including neurohormonal antagonists or device implantation [11]. Machine learning algorithms and network analysis serve as the computational core of precision cardiology, integrating genomic, proteomic, imaging and clinical data to generate models that forecast disease onset, progression and treatment response. On the therapeutic side, pharmacogenomic testing, for example genotyping for CYP2C19 to guide antiplatelet selection or screening for SLCO1B1 to reduce the risk of statin myopathy, translates laboratory discoveries into individualized drug choice and dosing [12]. Together, these modalities form a unified platform that enhances risk assessment, diagnosis and personalized therapy in modern cardiovascular medicine.

## Current clinical applications:

Precision medicine is increasingly woven into clinical cardiology workflows to enhance risk assessment, diagnostic precision and therapeutic decision making. Polygenic risk scores derived from genome wide association study data are now employed in select high risk populations to identify individuals with elevated lifetime risk of coronary artery disease, informing earlier preventive measures and more aggressive lipid management [13]. Inherited cardiac conditions such as hypertrophic cardiomyopathy and long QT syndrome routinely undergo targeted genetic testing panels to confirm diagnosis, guide family screening and tailor surveillance protocols. Integration of transcriptomic and proteomic biomarkers into clinical practice remains nascent but has shown promise in stratifying heart failure phenotypes, predicting disease progression and identifying patients likely to benefit from advanced therapies such as left ventricular assist devices. Pharmacogenomic insights have begun to alter prescribing patterns for common cardiovascular drugs [14]. Testing for CYP2C19 variants to guide antiplatelet selection after percutaneous coronary intervention reduces rates of stent thrombosis and bleeding by identifying patients with impaired clopidogrel metabolism who derive greater benefit from alternative P2Y12 inhibitors. Similarly, SLCO1B1 genotyping to assess statin uptake can predict risk of myopathy and enable dose adjustments or drug substitution to improve tolerability. In heart failure, emerging data suggest that genetic variants in adrenergic and renin angiotensin pathways may one day inform individualized choice and dosing of beta blockers and angiotensin converting enzyme inhibitors [15]. Advanced imaging phenotypes are also entering precision cardiology paradigms. Strain based echocardiography and tissue characterization by cardiac magnetic resonance provide detailed functional and structural metrics that complement molecular profiles in defining disease endotypes. For example, patients with non-ischemic cardiomyopathy exhibiting a fibrosis predominant signature on magnetic resonance may be steered toward early device therapy, while those with inflammatory expression profiles might benefit from immunomodulatory trials. These applications are supported by clinical registries that link imaging, genomic and outcome data to refine risk models and guide real world implementation [16]. Collectively, these current applications illustrate how precision cardiology is moving from concept to clinic, with measurable impacts on patient stratification, safety and outcomes.

## Biomarkers and predictive markers:

Biomarkers lie at the heart of precision cardiology by providing measurable indicators of disease susceptibility, progression and treatment response. Genetic markers such as pathogenic variants in the MYH7 MYBPC3 and LMNA genes offer definitive diagnosis and prognostic information in inherited cardiomyopathies, while polygenic risk scores encompassing thousands of common variants quantify an individual's predisposition to atherosclerotic disease beyond traditional clinical risk factors [17]. Circulating proteins including high sensitivity troponin N terminal pro B type natriuretic peptide and galectin 3 have been repurposed as molecular endophenotypes that stratify heart failure phenotypes, predict hospitalisation risk and monitor therapeutic efficacy. Emerging omics based markers such as microRNAs extracellular vesicle cargo and metabolomic signatures are under investigation for their ability to detect subclinical myocardial injury, distinguish ischemic from non-ischemic etiologies and anticipate adverse remodeling [18]. Imaging biomarkers complement molecular assays by capturing functional and structural changes in real time. Measures of myocardial strain extracellular volume fraction and late gadolinium enhancement provide quantitative surrogates of contractile dysfunction fibrosis and scar burden that correlate with arrhythmic risk and guide device therapy decisions [19]. Integration of these imaging phenotypes with molecular profiles through machine learning enhances predictive accuracy and reveals novel patient endotypes that respond differentially to therapies. Pharmacogenomic markers such as CYP2C19 genotype for clopidogrel metabolism and SLCO1B1 variants for statin intolerance have demonstrated clinical utility by informing antiplatelet selection and statin dosing, thereby reducing adverse events [20]. Future efforts will focus on multiplexed biomarker panels and dynamic sampling using serial blood or imaging assessments to enable adaptive treatment strategies and real time risk recalibration.

## Challenges and limitations:

The translation of precision medicine into routine cardiology practice is hindered by multiple interrelated challenges. Data integration remains a major obstacle because genomic, proteomic, imaging and electronic health record systems often operate in silos with incompatible data formats and variable quality control standards, making comprehensive multi modal analyses difficult to implement. The cost and logistical complexity of high throughput assays and advanced imaging protocols restrict their availability to specialized centers and exacerbate disparities in access, particularly in resource limited settings [21]. Regulatory and reimbursement frameworks have not yet adapted to accommodate evolving computational diagnostics and personalized therapeutic algorithms, leading to uncertainty among clinicians and payers. Ethical and privacy concerns regarding genetic data use and sharing further complicate consent processes and may erode patient trust. In addition, many machine learning models and polygenic risk scores have been developed and validated in predominantly European ancestry cohorts, raising questions about generalizability and the potential to worsen health inequities [22]. Finally, the lack of standardized clinical guidelines and prospective outcome data makes it challenging for practitioners to interpret molecular and computational findings and to integrate them into decision making, slowing the adoption of precision cardiology approaches across diverse practice environments.

## Future directions and emerging trends:

The future of precision cardiology lies in the convergence of increasingly sophisticated molecular assays, real time data analytics and seamless clinical integration. Single cell and spatial transcriptomic technologies promise to resolve the cellular heterogeneity of diseased myocardium and vascular tissue, uncovering novel pathogenic cell states and therapeutic targets. Advances in wearable and implantable sensors will generate continuous physiologic datasets that, when combined with genomic and proteomic profiles, enable dynamic risk prediction and preemptive intervention. Artificial intelligence frameworks trained on multi institutional, multi ancestry cohorts will improve the accuracy and equity of predictive models, facilitating their deployment in diverse healthcare settings. In the therapeutic realm, gene editing tools such as CRISPR Cas systems are being explored for *in vivo* correction of pathogenic variants underlying inherited cardiac disorders, while RNA based therapies offer the potential to modulate disease associated transcripts without permanent genome alteration. Integration of patient derived organoids and engineered heart tissues into drug screening platforms will accelerate the discovery of individualized treatment regimens and reduce reliance on animal models. Finally, the establishment of global precision cardiology consortia and standardized data sharing infrastructures will be critical to validate novel biomarkers, harmonize analytic pipelines and develop evidence based guidelines that translate scientific breakthroughs into improved patient outcomes across all populations.

## Conclusion:

The integration of multi omic profiling, advanced imaging, and computational analytics is reshaping cardiovascular care by enabling personalized risk assessment and targeted therapies. Overcoming barriers in data interoperability, cost, and equity will be essential to broaden the impact of precision cardiology. Continued collaboration across clinical, technological, and regulatory domains will drive the translation of these innovations into routine practice and improved patient outcomes.
